# Supplementary material for: Understanding and countering the spread of conspiracy theories in social networks: Evidence from epidemiological models of Twitter data
Source: PLoS One. 2021 Aug 12;16(8):e0256179. doi: 10.1371/journal.pone.0256179 (PMC8360523; doi:10.1371/journal.pone.0256179)
Supplement: S1 Table — (PDF) [file pone.0256179.s001.pdf]

**Parameter**	**Meaning**	**Guess**
β	infection rate	0.2
α	recovery rate	0.2
$\frac{1}{\alpha}$	infection period	5
$\mathcal{R}_0 = \frac{\beta}{\alpha}$	basic reproduction number	1
$S(0)$	initial susceptible	$n = 5611$
$I(0)$	initial infectious	1
$R(0)$	initial removed	$4n = 22444$
$N = S(0) + I(0) + R(0)$	population size	$5n + 1 = 28056$
