# Supplementary material for: Understanding and countering the spread of conspiracy theories in social networks: Evidence from epidemiological models of Twitter data
Source: PLoS One. 2021 Aug 12;16(8):e0256179. doi: 10.1371/journal.pone.0256179 (PMC8360523; doi:10.1371/journal.pone.0256179)
Supplement: S2 Table — (PDF) [file pone.0256179.s002.pdf]

**Model**	δ	γ	ζ	**Total Number of Infections**	**Total Number of Prevented Infections**	IP_f
basic	Inf	0	0	5435	0	0.770
fact-checking	14	0.01	0	4486	949	0.635
	14	0.03	0	2563	2871	0.363
	14	0.05	0	1128	4307	0.160
	42	0.01	0	4900	535	0.694
	42	0.03	0	3898	1537	0.552
	42	0.05	0	3039	2396	0.430
	59	0.01	0	5124	311	0.726
	59	0.03	0	4566	869	0.647
	59	0.05	0	4090	1345	0.579
deletion	14	0	0.06	5091	343	0.721
	14	0	0.12	4720	714	0.669
	14	0	0.25	3819	1616	0.541
	42	0	0.06	5097	337	0.722
	42	0	0.12	4736	699	0.671
	42	0	0.25	3880	1555	0.549
	59	0	0.06	5121	314	0.725
	59	0	0.12	4793	642	0.679
	59	0	0.25	4057	1378	0.575
mixed	14	0.01	0.06	4041	1393	0.572
	14	0.01	0.12	3555	1880	0.503
	14	0.01	0.25	2355	3080	0.333
	14	0.03	0.06	1990	3445	0.282
	14	0.03	0.12	1438	3996	0.204
	14	0.03	0.25	560	4875	0.079
	14	0.05	0.06	757	4678	0.107
	14	0.05	0.12	494	4941	0.070
	14	0.05	0.25	203	5232	0.029
	42	0.01	0.06	4524	911	0.641
	42	0.01	0.12	4123	1312	0.584
	42	0.01	0.25	3193	2242	0.452
	42	0.03	0.06	3477	1957	0.492
	42	0.03	0.12	3048	2387	0.432
	42	0.03	0.25	2150	3285	0.304
	42	0.05	0.06	2632	2803	0.373
	42	0.05	0.12	2242	3192	0.318
	42	0.05	0.25	1524	3911	0.216
	59	0.01	0.06	4802	633	0.680
	59	0.01	0.12	4469	966	0.633
	59	0.01	0.25	3742	1693	0.530
	59	0.03	0.06	4242	1193	0.601
	59	0.03	0.12	3918	1517	0.555
	59	0.03	0.25	3247	2188	0.460
	59	0.05	0.06	3780	1655	0.535
	59	0.05	0.12	3479	1956	0.493
	59	0.05	0.25	2883	2552	0.408
