# Supplementary material for: Understanding and countering the spread of conspiracy theories in social networks: Evidence from epidemiological models of Twitter data
Source: PLoS One. 2021 Aug 12;16(8):e0256179. doi: 10.1371/journal.pone.0256179 (PMC8360523; doi:10.1371/journal.pone.0256179)
Supplement: Attachment — Submitted filename: Response to reviewers.pdf [file pone.0256179.s003.pdf]

Point-By-Point Reply to Editor's Comments

Editor Point P 0.1 — Thank you for submitting your manuscript to PLOS ONE. After careful consideration, we feel that it has merit but does not fully meet PLOS ONE's publication criteria as it currently stands. Therefore, we invite you to submit a revised version of the manuscript that addresses the points raised during the review process.

Both reviewers greatly appreciated the work and found merit in it. At the same time, they highlighted areas where the manuscript could be improved. These mainly revolve around providing additional details, explanations, and discussion on the adopted methods. Please, do take the thorough reviewers' comments in great consideration when preparing your revision.

Reply: Thank you for the positive evaluation of our manuscript and for the invitation to submit a revised version of the manuscript.

Editor Point P 0.2 — Please ensure that your manuscript meets PLOS ONE's style requirements, including those for file naming.

Reply: Thank you for reminding us that the manuscript needs to meet PLOS ONE's style requirements. We have checked the manuscript and fixed a few minor issues: removed the postal/ZIP in the author affiliation 1 and removed the S1 Table (S2 Table in the revised version of the manuscript) from the manuscript. We have also made sure that our LaTeX template and the file naming are up-to-date. We also implemented a few small, formal corrections in webpage references (we now use the LaTeX "lastcheched" variable to indicate when the webpage was cited instead of adding it to the "year" variable). Please let us know in case there are other issues related to PLOS ONE's style requirements.

Editor Point P 0.3 — Please upload a copy of Supporting Information Table 1 which you refer to in your text on page 10.

Reply: Thank you for pointing out that the S2 Table needs to be uploaded separately. As stated in P 0.2 we removed S2 Table from the manuscript and uploaded a copy of it. Please note that the numbering of tables has changed in this revision.

Point-By-Point Reply to Reviewers' Comments

Reviewer #1

Reviewer Point P 1.1 — For example, it is not clear how the countermeasure here considered would work in a real setting and how they would produce the specific result claimed (bringing an individual from one compartment to another).

Reply: Thank you for pointing out this very important point. The question of how the two countermeasures introduced in the manuscript would work in a real setting depends in part on the respective social network. As stated in the Introduction section of the manuscript, social networks have proposed and introduced different strategies for how to address the occurrence of misinformation. Facebook, for instance, states that fact-checking performed by third-party fact-checkers is a condition for taking specific countermeasures, e.g. reduced distribution distribution of problematic posts or misinformation labels. The conceptualization of fact-checking in our manuscript is slightly different, as we understand fact-checking in a more preventive fashion, meaning that a susceptible

individual becomes "immune" by being exposed to true facts before he/she gets in contact with the specific misinformation. Twitter, on the other hand, states that they rely on misinformation labels as well, but also remove problematic tweets if they evidently transport harmful content. In response to this comment, we revised the Introduction section of our manuscript in order to clarify how fact-checking and deletion are applied in the respective networks. In this regard, we added another reference to underpin that tweet deletion is explicitly noted as a countermeasure by Twitter: Roth Y, Pickles N. Updating our approach to misleading information. In:Twitter Blog. 2020 May 11 [Cited 2021 June 2];Available from:`https://blog.twitter.com/en_us/topics/product/2020/updating-our-approach-to-misleading-information.html`.

With respect to the second part of your question, the assumption that the countermeasures exclusively work in a manner as introduced in the manuscript is very strict, and it is indeed reasonable to assume that they probably do not fully represent a real situation. However, we hold that even if the models are simplistic, they may reflect good starting points for more refined models. We revised the Discussion section in order to consider these points in greater detail.

Reviewer Point P 1.2 — Moreover, in the Parameter Identification paragraph, I think the concept of Incidence should be defined and contextualized better as well as the variable n and also it is understandable that the time unit is a single day since the data includes the frequency of each hashtag per day, but never explicitly stated.

Reply: We appreciate your point that neither the concept "Incidence" nor the variable n is contextualized appropriately in this or earlier paragraphs. Please note that as we intended to introduce a model which is defined independently from the underlying time scale (e.g. hours, days, weeks, ...) and suitable for different social networks, we deliberately did not precisely define these variables at this stage of the manuscript. We acknowledge that this may be confusing, and have revised the Data paragraph of our manuscript to clarify what "Incidence" and n mean in this context. This clarifies that the underlying time unit is indeed a single day in the context of the present study, but please note that the model definition operates on arbitrary time units.

Reviewer Point P 1.3 — In my opinion also the Data section should be revised. All the information seems to be present, but the discourse is a bit unclear: I do not know if it is necessary to explain why in the end the choice went on the hashtag data set instead of full tweets, but maybe I would focus more on the description of the data and how despite limitations they may be enough to understand the underlying phenomena.

Reply: Thank you for providing suggestions on how the Data section can be improved. We agree with you and have shortened some paragraphs in the manuscript. In particular, we removed the paragraph about how the full tweets were recorded and why they could not be used in the end. However, we believe that our approach to fully download the tweets, allowing to map the spread of misinformation on an individual/user level, is nevertheless a worthwhile strategy for further studies. We therefore added a note to the Discussion section, where we described our approach and why future studies may should rely on such data. We also revised the Data section in order to clarify the nature of our data and their potential to capture the spread of conspiracy theories through Twitter to an acceptable degree. We added two references to underpin our view that the data is appropriate to enhance understanding of the underlying phenomena: Tekumalla R, Banda JM. Social Media Mining Toolkit (SMMT). Genomics & Informatics. 2020;18(2). `doi:10.5808/GI.2020.18.2.e16`. and Lamsal R. Design and analysis of a large-scale COVID-19 tweets dataset. Applied Intelligence. 2021;51(5, SI):2790–2804.`doi:10.1007/s10489-020-02029-z`.

Reviewer Point P 1.4 — I also think that in the description of the Results some improvements may come from a more detailed description of the values from the real data to the values

estimated by the models, especially when considering plots and tables, which are not thoroughly described within the text or the caption. Maybe some of the key evidence from the tables/figures in terms of numerical values may be added to the text and not left to the reader to understand. For example, in figure 3A some of the percentages of IPf may be reported and compared across models and to the baseline value, which I also think should be stated at the beginning of this section and not after the description of all three extensions, to give the reader a chance to understand the differences from the extended models without having to go back.

Reply: Thank you for pointing out that the Results section can be improved by adding a more detailed description of how the observed data corresponds to the model predictions. We addressed this concern in the revised manuscript by incorporating more details and numerical values (as shown in the figures/tables) in the text. With respect to Fig. 3, we added further details and explanations to clarify what is shown in this Figure. We also enhanced the figure caption of Fig. 3 to better guide the reader through panel A and B.

Reviewer Point P 1.5 — For what concerns the Discussion section, I think general conclusions and limitations are well assessed, however I think some points should have been presented also in the results section and not here for the first time, for example things such as the models failing to capture the second smaller peak, how smaller is the peak with respect to the first one...

Reply: Thank you for stating that the conclusions and limitations of the Discussion section are well assessed. We agree with you that the inability of the model to account for the second peak should already be presented in the Results section. We revised the Results sections accordingly. However, we kept the point in the Discussion section as well, as we believe that future studies should put effort into developing models capable of capturing the reappearance of misinformation in social networks.

Reviewer #2

Reviewer Point P 2.1 — Reference [1] is not so enough to support the sentence after which it is mentioned. I think something like Zarocostas, John. "How to fight an infodemic." The lancet 395.10225 (2020): 676. would be better.

Reply: Thank you for pointing out that Reference [1] is insufficient to support the sentence after which it is mentioned. We agree with you and picked up your reference appreciatively.

Reviewer Point P 2.2 — The authors do not specificy what they mean by incidence and cumulative incidence (I_{cum}). I guess it is the total outreach of posts/hashtags.

Reply: Thank you for pointing out that the incidence and cumulative incidence (I_{cum}) are not specified appropriately. This point is partly addressed by P 1.2. Indeed, the Incidence refers to the number of "suspect" (see Table 2 in the manuscript) hashtags per day, and cumulative incidence (I_{cum}) is the cumulated number of "suspect" hashtags up to a specific day. We revised the Data section in order to clarify what is meant by Incidence in this context.

Reviewer Point P 2.3 — The authors should provide further details regarding the estimation of the SIR parameters (especially N whose inference seems to be crucial for the results of the model)

Reply: Thank you for suggesting that the estimation of the SIR parameters should be explained in greater detail. We revised the paragraph in question and added some details on how the parameters were identified. We added another supporting information table (S1 Table) to explicitly provide our initial guesses for parameters to be optimized over. We also clarified which optimization algorithm we used and added the corresponding reference: Nelder JA, Mead R. A Simplex Method for Function Minimization. The Computer Journal. 1965;7(4):308–313. doi:10.1093/comjnl/7.4.308.

Indeed, the identification of N is very relevant for the models presented in the manuscript. We performed a control analysis where we artificially increased the population size to approx. 3 times the population size reported in the manuscript ($N = 9999$). With respect to the basic SIR model, the model fit and the predictions of the Incidence did not decrease significantly, but the initial size of the R ($R(0)$) compartment increased substantially, meaning that, according to this model, the majority of the population was not susceptible to the conspiracy theory. With respect to the extended SIR models, we found that the Incidence predictions did not change significantly for the tweet-deletion model and changed slightly for the fact-checking model (in a manner that an early response is even more effective than in the model shown in the manuscript). In general, the predictions of the extended SIR models seem to be relatively robust towards changes in the population and compartment sizes. For the moment, we have refrained from including this additional information in the SI of our manuscript, but we would be happy to add it if so advised by the editor.

Reviewer Point P 2.4 — Do the authors expect different results by relaxing the assumption of full mixing of the SIR model? Social networks are generally sparse while (if I am not wrong) the SIR in the version discussed in the paper doesn't consider this aspect. (see for instance The echo chamber effect on social media M Cinelli, GDF Morales, A Galeazzi, W Quattrociocchi, M Starnini Proceedings of the National Academy of Sciences 118 (9))

Reply: Thank you for mentioning this very important point. We perfectly agree with you that social networks are - in general - sparse. Incorporating this point into the epidemiological model very likely helps to better understand the true spread of misinformation/conspiracy theories in social networks. As our models should be seen as starting points for more refined analyses, we did not considered heterogeneous contact patterns in our manuscript. Speculatively, the SIR model presented in the manuscript inherently represents a homogeneous subgroup of the population: The majority of the population was - according to the model - susceptible to the conspiracy theory. Even though this is not realistic, it might take account for the possibility of a minority/subgroup on Twitter which is susceptible to conspiracy theories, and which is relatively homogeneous in that respect. However, we appreciate your point and complemented the Discussion paragraph addressing the problems of the simple SIR model accordingly and also added another reference in order to point towards approaches how to explicitly model heterogeneity in contact patterns: Bansal S, Grenfell BT, Meyers LA. When individual behaviour matters:homogeneous and network models in epidemiology. JOURNAL OF THE ROYALSOCIETY INTERFACE. 2007;4(16):879–891. doi:10.1098/rsif.2007.1100. We also modified the Results section slightly and incorporated your reference there appreciatively.

Reviewer Point P 2.5 — The correct reference for Ref 6 is: The COVID-19 Social Media Infodemic [...] Scientific Reports volume 10, Article number: 16598 (2020)

Reply: Thank you for pointing out that Ref 6 is incorrect. We revised the reference in question.
